# An updated checklist of *Begonia* (Begoniaceae) in Laos, with two new species and five new records

**DOI:** 10.3897/phytokeys.138.46718

**Published:** 2020-01-10

**Authors:** Hong-Bo Ding, Mya Bhone Maw, Bin Yang, Somsanith Bouamanivong, Yun-Hong Tan

**Affiliations:** 1 Southeast Asia Biodiversity Research Institute & Center for Integrative Conservation, Xishuangbanna Tropical Botanical Garden, Chinese Academy of Sciences, Menglun, Mengla, Yunnan 666303, China Xishuangbanna Tropical Botanical Garden, Chinese Academy of Sciences Yunnan China; 2 Center of Conservation Biology, Core Botanical Gardens, Chinese Academy of Sciences, Menglun, Mengla,Yunnan 666303, China Core Botanical Gardens, Chinese Academy of Sciences Yunnan China; 3 University of Chinese Academy of Sciences, Shijingshan District, Beijing 100049, China University of Chinese Academy of Sciences Beijing China; 4 Ecology Division, Biotechnology and Ecology Institute, Ministry of Science and Technology, P.O. Box: 2279, Vientiane Capital, Lao PDR Ministry of Science and Technology Vientiane Lao People's Democratic Republic

**Keywords:** sect. *Parvibegonia*, sect. *Reichenheimia*, taxonomy, new records

## Abstract

Two new species of *Begonia* L. (Begoniaceae), *B. laotica* (sect. Parvibegonia) and *B. hypoleuca* (sect. Reichenheimia), from north Laos are described and illustrated. *Begonia augustinei*, *B. dryadis*, *B. lancangensis*, *B. sizemoreae* and B. sillentensis subsp. mengyangensis were newly recorded taxa in Laos. Furthermore, an updated checklist of *Begonia* of Laos is also compiled.

## Introduction

*Begonia*Linnaeus (1753: 1056) is the sixth largest genera of angiosperms and the number of accepted species of *Begonia* currently stands at 1947 (Hughes et al. 2015) and is likely to rise to well over 2000 (Moonlight et al. 2018; Tian et al. 2018). According to the annotated checklist of south east Asia *Begonia* by Hughes (2008) and recent taxonomic publications (Hughes 2007; Newman et al. 2007; De Wilde et al. 2011; Averyanov and Nguyen 2012; Souvannakhoummane et al. 2016, 2018; Yang et al. 2018; Averyanov et al. 2019), 19 species of *Begonia* have been described from Laos, so far.

As part of the botanical inventory of China-Laos transboundary biodiversity conservation, we carried out floristic surveys in the Phou Hin Phee National Biodiversity Conservation Area, Oudomxay Province, the Nam Ha National Biodiversity Conservation Area in Luang Namtha Province and the Phou Dean Din National Bio-Diversity Park, Phongsaly Province of northern Laos. During the fieldwork from 2018 to 2019, some interesting species of *Begonia* have been collected. After reviewing the literature and herbarium specimens, we described and illustrated two species as new to science and five species as new records for the flora of Laos. We provided an updated total of 26 species for the *Begonia* flora of Laos.

*Begonia laotica* is classified under B. sect. Parvibegonia A. de Candolle (1859: 136) according to its small and tuberous habitat, axile placentation, 2-locular ovary, 2 styles and fruit with 3 unequal wings (Doorenbos et al. 1998; Moonlight et al. 2018). *Begonia hypoleuca* belongs to B. sect. Reichenheimia (Klotzsch, 1855: 174) A. de Candolle (1864 385), based on the morphological characteristics, including 2 tepals in male flowers, axile and entire placentation, 3-locular ovary, 3 styles and fruit with 3 wings (Doorenbos et al. 1998; Moonlight et al. 2018).

## Material and methods

Measurements and morphological character assessments of the new species have been examined, based on fresh materials and dried specimens. They have been compared with morphologically similar species by affinities inferred using descriptions (Ku et al. 2007; Camfield and Hughes 2018) and type specimens in herbaria (BM, E, K, B, KUN, P, HITBC, YUNU, LE and FOF). Protologues and images of type specimens were gathered from JSTOR Global Plants (http://plants.jstor.org).

## Taxonomic treatments

### Begonia laotica

Taxon classificationPlantaeCucurbitalesBegoniaceae

Y.H.Tan & H.B.Ding sp. nov.

39CCE066-98C3-5782-8901-47469E69080B

urn:lsid:ipni.org:names:77204211-1

Figure 1

sect. Parvibegonia A. de Candolle

#### Diagnosis.

The new species is mostly similar to *B. josephii* A. de Candolle (1859: 126) in its peltate, stemless, tuberous habit, but significantly differs by fleshy reddish bristles at the petiole apex (vs. without), ovary 2-locular (vs. 3-locular) and styles 2 (vs. 3).

#### Type.

Laos. Oudomxay Province, Maung Xai, Phou Hin Phee National Biodiversity Conservation Area, 20°43'18.69"N, 102°08'47.97"E. 1378 m elev., 25 October 2018, flowering, *Y.H. Tan & H.B. Ding L0827* (holotype: HITBC!; isotypes: HNL!).

#### Description.

Monoecious herb, stemless, tuberous, 18−25 cm high, tuber small, subglobose, 12−15 mm in diameter. ***Stipule***: broadly lanceolate, 2−3 mm long, membranous, sparsely glandular hairy, margin fringed by hairs, deciduous. ***Leaf*: *petiole*** 6−15 cm long, glabrous or sparsely puberulous; blade ovate, nearly symmetric, 9.2−13.5 × 5.8−11 cm, peltate, base rounded, apex acuminate, margin slightly denticulate, with sparse short hairs, upper surface green, glabrous, lower surface paler, venation palmate-pinnate, tomentose on the veins, fleshy reddish bristles at the petiole apex, thinly succulent in life, thinly papery when dried. ***Inflorescence***: axillary, terminal cyme; peduncle puberulent, 7.2−10.9 cm long, branching 2−3 times; bracts narrowly ovate, ca. 4.0 × 2.5 mm, membranous, reddish line along the middle, slightly glandular hairy, margin fringed by glandular hairs. ***Staminate flower***: pedicel 1.1−1.8 cm, membranous, covered with glandular hairs, tepals 4, pink, unequal, outer 2, ovate, 10−13 × 7−17 mm, glandular hairs on the outer surface, margin entire, inner 2, elliptic, 7−10 × 2−4 mm, glabrous, margin entire; stamens 15−20, anther obovate, ca. 1 mm. ***Pistillate flower*** (young): pedicel 0.9−1.2 cm long, membranous, glandular hairy, tepals 5, pink, unequal, outer 2, ovate, 4−6 × 3−4 mm, inner 2, elliptic, 3−5 × 1−3 mm, glandular hairs on the outer surface, especially at the base; ***ovary*** 2-locular, placentae axial, placentae 2 per locule, ***styles*** 2, stigmas bifid with twisted bands, highly convolute, golden yellow. ***Capsule*** nodding, obovate, pinkish tomentose, unequally 3-winged, abaxial wing oblong, ca. 13 mm long, lateral wings shorter, ca. 3 mm long.

#### Phenology.

Flowering in October–November and fruiting in December.

#### Distribution.

Endemic to the type locality, Oudomxay Province, Maung Xai, Phou Hin Phee National Biodiversity Conservation Area, Laos.

#### Ecology.

Restricted to the shaded base of limestone cliff at ca. 1378 m elevation.

#### Etymology.

The specific epithet ‘*laotica*’ refers to the type locality in Laos.

#### Conservation status.

Data Deficient (DD) (IUCN 2017). The new species was only collected from the limestone cliff of Hin Phee National Biodiversity Conservation Area. Due to its remote habitat, we suggested that the species may not suffer from strong human disturbance. According to the available information, we proposed the preliminary conservation status of this new species as Data Deficient (DD) (IUCN 2017).

#### Notes.

*Begonia laotica* is phenotypically most similar to *B. josephii* in its tuberous habit, ovate and peltate leaves, but it can easily be distinguished by its glabrous upper leaf surface (vs. hispid all over), fleshy reddish bristles at the petiole apex (vs. without), bracts narrowly ovate and fringed by glandular hairs (vs. lanceolate and glabrous), male flowers pedicels with glandular hair (vs. glabrous), outer tepals ovate (vs. broadly obovate), inner tepals elliptic (vs. spathulate), anther obovate (vs. ellipsoid), female flowers pedicels with sparsely glandular hair (vs. glabrous), glandular hair on the outer surface (vs. glabrous), ovary 2-locular (vs. 3-locular) and styles 2 (vs. 3). This new species also shares similar characteristics with *B. subperfoliata* Parish ex Kurz (1873: 81) in tuberous habit and peltate leaves, but it can easily be distinguished by its ovary 2-locular (vs. 3-locular) and styles 2 (vs. 3).

**Figure 1.**
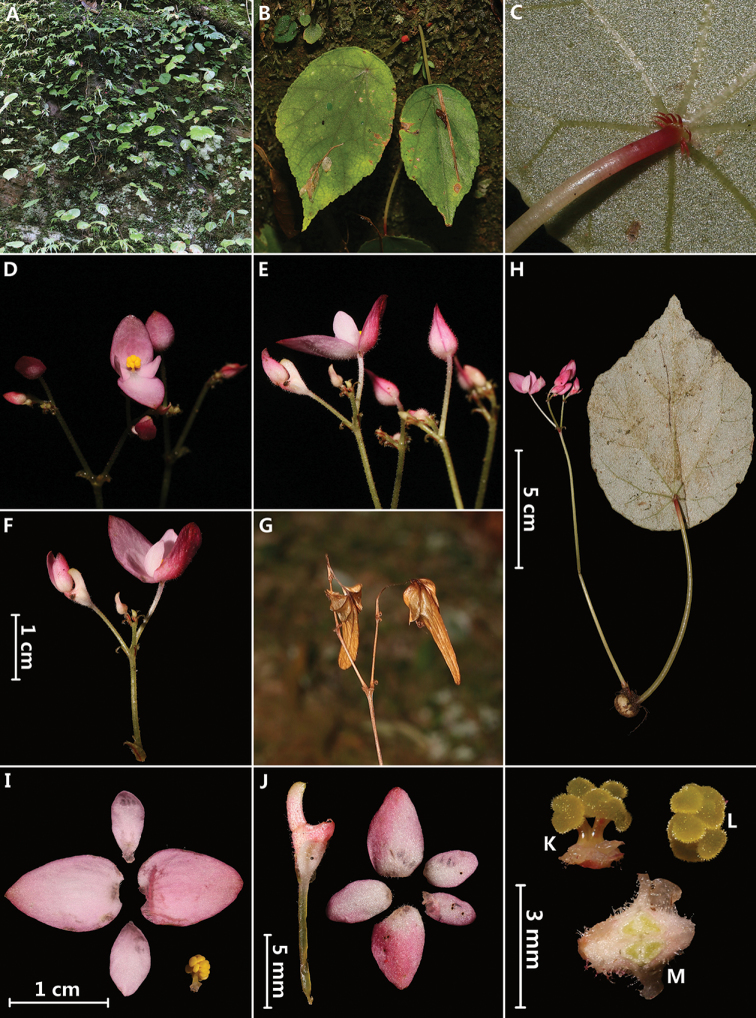
*Begonia laotica* Y.H.Tan & H.B.Ding, sp. nov. **A** habitat **B** leaf blade adaxially **C** leaf blade abaxially (showing fleshy reddish bristles at the petiole apex) **D** flowers (front view) **E** flowers (lateral view) **F** inflorescence **G** mature fruits **H** habit **I** staminate flower **J** pistillate flower (young) **K** stamens (lateral view) **L** stamens (front view) **M** cross section of ovary. Photographed by H.B. Ding.

### Begonia hypoleuca

Taxon classificationPlantaeCucurbitalesBegoniaceae

Y.H.Tan & H.B.Ding sp. nov.

A2CD853A-A49A-5DC9-AE9D-8D3E85BA040D

urn:lsid:ipni.org:names:77204213-1

Figure 2

sect. Reichenheimia (Klotzsch) A. de Candolle

#### Diagnosis.

The new species is mostly similar to *B. henryi*Hemsley (1887: 322), but differs by its glabrous leaves, petiole with linear red dots and pubescent, slightly denticulate leaf margin and fewer stamens.

#### Type.

Laos. Luang Namtha Province, Nam Ha National Biodiversity Conservation Area, Nam O. Village, 20°04'48.16"N, 101°06'58.46"E. 656 m elev., 24 October 2018, *Y.H. Tan*, *B. Yang*, *H.B. Ding & X.D. Zeng L0792* (holotype: HITBC!).

#### Description.

Monoecious herb, stemless, tuber small, globose with fibrous roots, 10−13 mm in diameter. ***Stipule***: caducous. ***Leaf***: petiole 7−10 cm long, succulent, with reddish linear dots, sparsely pubescent or subglabrous. ***Blade*** slightly triangular ovate or broadly ovate, 6−12 × 5−8.5 cm, base cordate, apex acuminate, margin slightly denticulate, upper surface green, lower surface paler or silver, glabrous, venation palmate-pinnate, midrib pale green, lateral veins 4−6 pairs. ***Inflorescence*** axillary, cymose, peduncle ca. 6 cm long, reddish, glabrous, branching 2−4 times, 2−6 male flowers, ca. 2 pistillate flowers, bracts caducous, ca. 2 × 1 mm. ***Staminate flower***: pedicel 1.0−1.2 cm long, membranous, reddish at the basal part and pink, glabrous, tepals 2, pink, margin entire, glabrous on both surfaces, broadly ovate, 12 × 10 mm, apex acute, base rounded; stamens 10−15, filaments 1.5−2 mm long, anthers 1−1.5 mm, ellipsoid, yellow. ***Pistillate flower***: pedicle ca. 1.8 cm long, reddish to pink, tepals unknown, ***ovary*** 3-locular, placentae axial, entire, ***styles*** 3. ***Capsule*** pale green, nodding, ellipsoid, dorsal wing enlarged, 7−10 mm long, two lateral wings, smaller, 2−3 mm long.

#### Phenology.

Flowering and fruiting in October–November.

#### Distribution.

The species is known only from the type locality, Luang Namtha Province, Nam Ha National Biodiversity Conservation Area, Laos.

#### Ecology.

The species was collected on shaded moist limestone rock surfaces of stone forest at 656 m elevation.

#### Etymology.

The specific epithet ‘*hypoleuca*’ refers to the lower surface of its paler or silver leaf.

#### Conservation status.

Data Deficient (DD). *Begonia hypoleuca* has been collected on only one occasion from the type locality. However, the type locality is located in the Nam Ha National Biodiversity Conservation Area and we did not discover any strong pressure on the species. Thus, the species preliminarily has been assigned in the Data Deficient (DD) category as being appropriate according to the guidelines for using the IUCN Red List Categories and Criteria (IUCN 2017).

#### Notes.

*Begonia hypoleuca* is most similar in morphological characteristics to *B. henryi* Hemsl. under the section Reichenheimia. It can, however, be distinguished by the following characteristics, including reddish linear red dots on petiole (vs. without), sparsely pubescent on petiole (vs. brown villous), slightly denticulate leaf margin (vs. crenate), glabrous leaves (vs. pubescent), leaf upper surface green (vs. green with dark green patches), lower surface pale green (vs. pale green with reddish patches), male flower tepals broadly ovate (vs. oblate-orbicular), apex acute (vs. rounded), stamens number fewer 10–15 (vs. up to 30).

**Figure 2.**
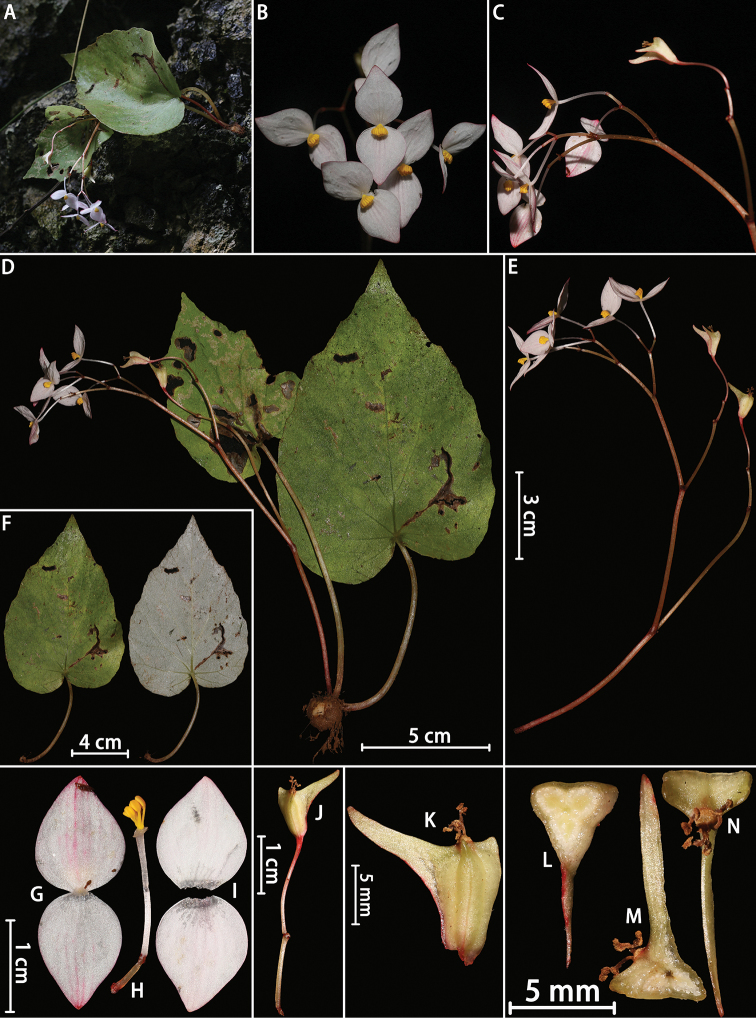
*Begonia hypoleuca* Y.H.Tan & H.B.Ding, sp. nov. **A** habitat **B** flowers (front view) **C** flowers (lateral view) **D** habit **E** inflorescence **F** leaves **G** tepals of staminate flower (back view) **H** stamens with peduncle **I** tepals of staminate flower (front view) **J** mature fruit with peduncle **K** mature fruit **L–N** serial cross sections of ovary. Photographed by H.B. Ding.

### New records for Laos

#### Begonia augustinei

Taxon classificationPlantaeCucurbitalesBegoniaceae

Hemsley

0540743C-05F1-5DB3-9CA7-8237772EF1F6

Figure 3 A–E

Begonia augustinei Hemsley, Gard. Chron 3 (28): 286. 1900; T.C. Ku in T.L. Wu (ed.), Fl. Reipubl. Popularis Sin. 52(1): 256. 1999; S.H. Huang & Y.M. Shui in C.Y. Wu (ed.), Fl Yunnan. 12: 213. 2006; T.C. Ku et al. in C.Y. Wu & P.H. Raven (eds), Fl. China 13: 163. 2007. Type: China, Yunnan Province, Simao, *A. Henry 12333A* (holotype: B100365120!).

##### Specimens examined.

Laos. Luang Namtha Province, Nam Ha National Biodiversity Conservation Area, Near Nalan Neua Village, 20°49'54.92"N, 101°19'24.85"E. 646 m elev., 13 October 2018, *Y.H. Tan*, *B. Yang*, *H.B. Ding & X.D. Zeng L0687* (HITBC; HNL); Oudomxay Province, Maung Xai, Phou Hin Phee National Biodiversity Conservation Area, 20°43'30.86"N, 102°08'42.39"E. 1288 m elev., 25 October 2018, *Y.H. Tan & H.B. Ding L0819* (HITBC; HNL); Oudomxay Province, Maung Xai, Phou Hin Phee National Biodiversity Conservation Area, 20°43'23.74"N, 102°08'42.84"E. 1224 m elev., 25 October 2018, *Y.H. Tan & H.B. Ding L0822* (HITBC).

##### Distribution.

China, Laos.

**Note.** This species is characterised by elongate rhizomes, extremely asymmetric leaves, base cordate to deeply cordate, margin irregularly serrulate (Hemsley 1900; Ku 1999; Huang and Shui 2006; Ku et al. 2007).

**Figure 3.**
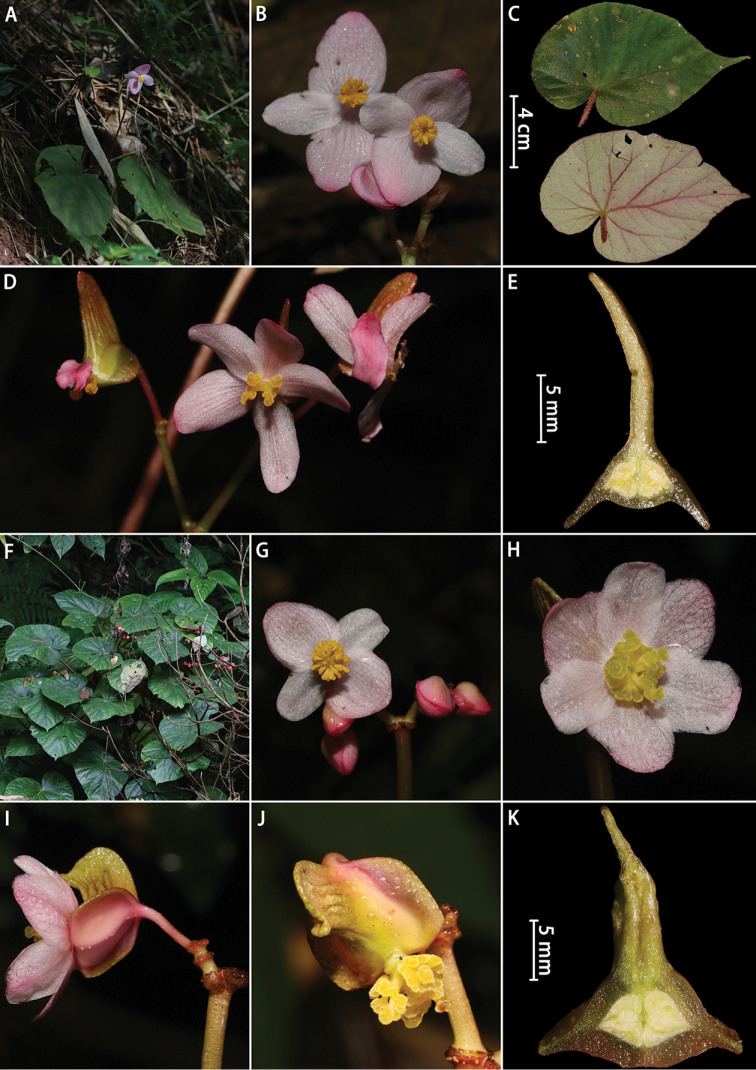
**A–E***Begonia augustinei* Hemsley: **A** habitat **B** staminate flowers (front view) **C** leaf blade (adaxially and abaxially) **D** pistillate flowers **E** cross section of ovary **F–K***Begonia dryadis* Irmscher: **F** habitat **G** staminate flowers (front view) **H** pistillate flower (front view) **I** pistillate flower (lateral view) **J** fruit **K** cross section of ovary. Photographed by H.B. Ding.

#### Begonia dryadis

Taxon classificationPlantaeCucurbitalesBegoniaceae

Irmscher

81FC1FA8-375E-5730-85CF-D6E2DF35CDE6

Figure 3 F–K

Begonia dryadis Irmscher, Not. Bot. Gard. Edinb. 21(1): 41. 1951; T.C. Ku in T.L. Wu (ed.), Fl. Reipubl. Popularis Sin. 52(1): 246. 1999; S.H. Huang & Y.M. Shui in C.Y. Wu (ed.), Fl Yunnan. 12: 215. 2006; T.C. Ku et al. in C.Y. Wu & P.H. Raven (eds), Fl. China 13: 171. 2007. Type: China, Yunnan Province, Kuen-ger, Che-li Hsien, 1100 m elev., October 1936, *C.W. Wang 79349* (holotype: KUN0370809).

##### Specimens examined.

Laos. Oudomxay Province, Maung Xai, Phou Hin Phee National Biodiversity Conservation Area, 20°43'18.52"N, 102°08'47.16"E. 1368 m elev., 30 March 2018, *Y.H. Tan*, *B. Yang*, *H.B. Ding & X.D. Zeng L0356* (HITBC; HNL); Oudomxay Province, Maung Xai, Phou Hin Phee National Biodiversity Conservation Area, 20°43'18.74"N, 102°08'47.84"E. 1364 m elev., 25 October 2018, *Y.H. Tan & H.B. Ding L0815* (HITBC).

##### Distribution.

Chian, Laos.

##### Note.

This species was first discovered and reported in the forest understorey, by streams in a valley of South Yunnan, China. The discovery of this species in Laos shows the geographical linkage between two type localities. The species is differentiated from the allied species by its short, stout rhizome and the presence of cauline leaves. (Irmscher 1951; Ku 1999; Huang and Shui 2006; Ku et al. 2007).

#### Begonia lancangensis

Taxon classificationPlantaeCucurbitalesBegoniaceae

S.H. Huang

5DBE6E2E-1674-5F66-807C-66FAF5E59F8D

Figure 4 A–C

Begonia lancangensis S.H. Huang, Acta Bot. Yunnan. 21(1): 13. 1999; S.H. Huang & Y.M. Shui in C.Y. Wu (ed.), Fl Yunnan. 12: 230. 2006; T.C. Ku et al. in C.Y. Wu & P.H. Raven (eds), Fl. China 13: 181. 2007. Type: China, Yunnan Province, Lancang Xian, Fazhan He, 1600 m elev., 1995, *Huang Suhua 95001* (holotype: YUNU).

##### Specimens examined.

Laos. Luang Namtha Province, Nam Ha National Biodiversity Conservation Area, Near Na Lun Village, 20°50'39.62"N, 101°19'41.46"E. 687 m elev., 23 March 2018, *Y.H. Tan*, *B. Yang*, *H.B. Ding & X.D. Zeng L0055* (HITBC).

##### Distribution.

China, Laos.

##### Note.

The species is characterised by its dioecious and erect stems with ovate or ovate-oblong leaves and fleshy berry-like fruits (Shui and Huang 1999, Ku et al. 2007). Additionally, *B. handelii* Irmscher (Irmscher 1921: 24) and *B. acetosella* Craib (Craib 1912: 153) have been discovered in the same locality. We suspect that this species may be a natural hybrid species of them.

**Figure 4.**
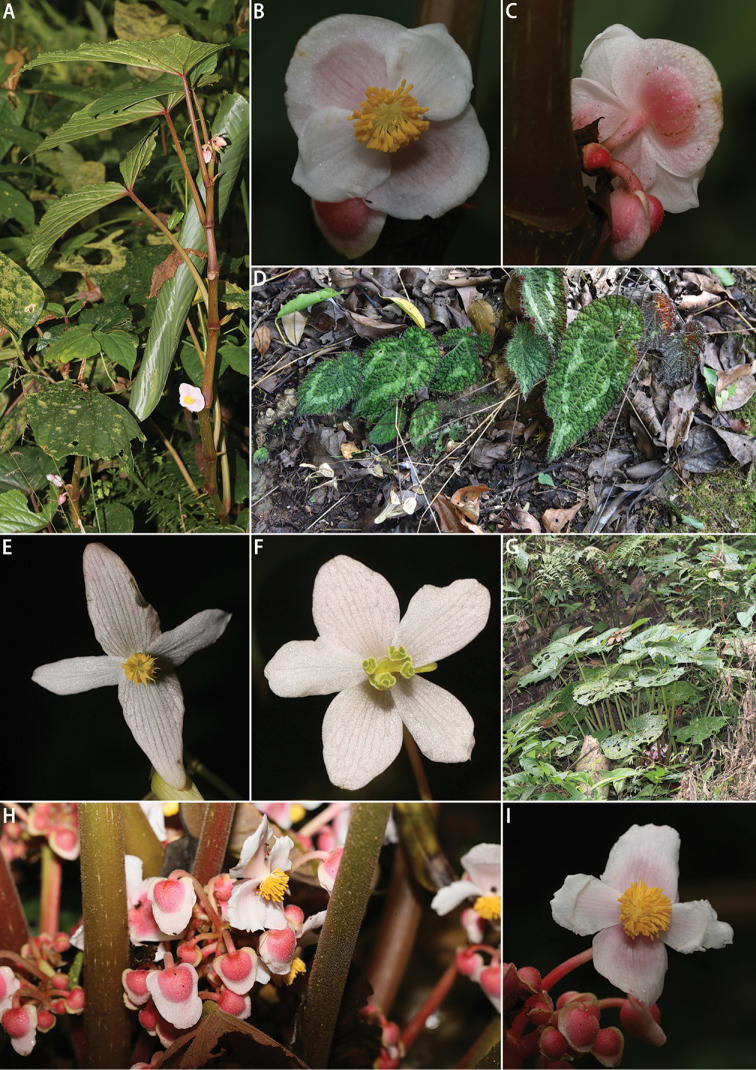
**A–C***Begonia lancangensis* S.H. Huang: **A** habitat **B** staminate flower (front view) **C** staminate flower (back view) **D–F***Begonia sizemoreae* Kiew: **D** habitat **E** staminate flower **F** pistillate flower **G–I**Begonia silletensis (A. DC.) C.B. Clarke subsp. mengyangensis M.C. Tebbitt & K.-Y. Guan: **G** habitat **H** inflorescence **I** staminate flower (front view). Photographed by H.B. Ding and Y.H. Tan.

#### Begonia sizemoreae

Taxon classificationPlantaeCucurbitalesBegoniaceae

Kiew

BE6B512B-D329-514C-9439-F92CFE5E1F6A

Figure 4 D–F

Begonia sizemoreae Kiew, in Gard. Bull. Singapore 56: 95. 2004; Kiew R. in Adansonia sér. 3, 29(2): 234. 2007. Type: Vietnam, Ba Vi National Park, Ha Tay Province, ca. 80 km west of Hanoi, Accession No. 20020399 cult. in Singapore Botanic Gardens ex Palm Hammock Orchid Estate, Miami, U.S.A. *R. Kiew 5304* (holotype: SING, isotype: HN).

##### Specimens examined.

Laos. Phongsaly Province, Phou Dean Din National Bio-Diversity Park, 21°46'02.39"N, 102°31'28.58"E. 1111 m elev., 8 April 2018, *Y.H. Tan*, *B. Yang*, *H.B. Ding & X.D. Zeng L0559* (HITBC).

##### Distribution.

Vietnam, Laos.

##### Note.

*Begonia sizemoreae* was originally described in north Vietnam. Now, its distribution in Laos has been confirmed. This species is very similar to *B. rex* Putz. (Putzey 1857: 141) from Assam, India, in its leaf shape, fine variegated leaves and flower structure, but it is distinct in its leaf margin with very hairy upper surface of the lamina with hairs 5–10 mm long and its conspicuous deep crimson tertiary venation in the centre and outer part of the lower leaf surface (Kiew 2004, 2007).

#### Begonia silletensis subsp. mengyangensis

Taxon classificationPlantaeCucurbitalesBegoniaceae

M.C. Tebbitt & K.Y. Guan

B79BF2E5-0B78-58CE-9E74-E3946B9A9363

Figure 4 G–I

Begonia silletensis (A. DC.) C.B. Clarke subsp. mengyangensis M.C. Tebbitt & K.-Y. Guan, Novon 12(1): 134. 2002; T.C. Ku et al. in C.Y. Wu & P.H. Raven (eds), Fl. China 13: 198. 2007. Type: China, Yunnan Province, Xishuangbanna, on the way from Puwen to Mengyang, at the bottom of valley in a wet area on the slope facing N, dense forest, 21 April 1957, *Sino-Soviet Union expedition 9633* (holotype KUN0533687!).

##### Specimens examined.

Laos. Luang Namtha Province, Nam Ha National Biodiversity Conservation Area, Near Na Lun Village, 20°50'34.97"N, 101°20'05.69"E. 632 m elev., 23 March 2018, *Y.H. Tan*, *B. Yang*, *H.B. Ding & X.D. Zeng* L0095 (HITBC; HNL).

##### Distribution.

India, China, Bangladesh, Myanmar and Laos.

**Note.** The species is characterised by its dioecious and prostrate stems with large broad leaves and fleshy berry-like fruits (Tebbitt and Guan 2002; Ku et al. 2007).

### An updated checklist of *Begonia* species in Laos

Begonia sect. Diploclinium

***Begonia adscendens*** C.B. Clarke

**Laos**: Champasak: *J.K. Munzinger 250* (L, P).

***Begonia hinnamnoensis*** Souvann & Lanors

**Laos**: Khammouan: *Lamxay et al. HNN227* (holotype HNL, isotypes FOF, E, SING).

***Begonia khammouanensis*** Souvann. & Lamxay

**Laos**: Khammouan: *Lamxay et al. HNN138* (holotype HNL, isotypes FOF, E, SING).

***Begonia modestiflora*** Kurz

**Laos**: Bassac: *Maxwell 97-1081* (L); Me-Kong: *C. Threl 2239* (BM, P).

***Begonia tatianae*** Aver.

**Laos**: Khammouan: *L. Averyanov*, *AL 820b.1* (holotype LE01049623), *L. Averyanov et al. AL820b* (paratype LE01049002).

***Begonia viscosa*** Aver. et H.Q. Nguyen

**Laos**: Vientiane: *L. Averyanov*, *P.V. The*, *CPC2438* (holotype LE, isotype CPC).

Begonia sect. Jackia

***Begonia cladotricha*** M.Hughes

**Laos**: Khammouan: *M.F. Newman et al. LAO985* (holotype E).

Begonia sect. Parvibegonia

***Begonia integrifolia*** Dalzell

**Laos**: Champasak: *J.F. Maxwell 97-1150* (CMU, L), *C. Thorel 2226* (P[2]); *C. Thorel 2226A* (P[2]).

***Begonia laotica*** Y.H. Tan & H.B. Ding

**Laos**: Oudomxay: *Y.H. Tan*, *H.B. Ding L0827* (holotype HITBC!, isotypes HNL!).

***Begonia namkadingensis*** C.-J. Yang, Souladeth & Tagane

**Laos**: Bolikhamxay: *Tagane S. et al. L1202* (holotype FOF, isotypes HAST, HNL, KYO), *Tagane S. et al. L960* (FOF, KYO, TAI).

***Begonia procridifolia*** Wall. ex A.DC.

**Laos**: *A.F.G. Kerr 21188* (K, P).

Begonia sect. Petermannia

***Begonia lamxayiana*** Souvann

**Laos**: Bolikhamxay: *V. Lamxay et al. VL2198* (holotype HNL).

Begonia sect. Platycentrum

***Begonia acetosella*** Craib

**Laos**: *E. Poilane 20721* (P); Luang Namtha: *Tan et al. L0050* (HITBC!, HNL!), *Tan et al.* L0083 (HITBC!); Oudomxay: *Tan et al.* L0202 (HITBC!, HNL!).

***Begonia augustinei*** Hemsl

**Laos**: Luang Namtha: *Tan et al. L0687* (HITBC; HNL); Oudomxay: *Tan et al. L0819* (HITBC; HNL), *Tan et al. L0822* (HITBC).

***Begonia dryadis*** Irmsch

**Laos**: Oudomxay: *Tan et al. L0356* (HITBC; HNL), *Tan et al. L0815* (HITBC).

***Begonia handelii*** var. ***handelii*** Irmsch.

**Laos**: Luang Namtha: *L.J. Ahnby 129* (E), *Tan et al. L0061* (HITBC; HNL); Phongsaly: *Tan et al. L0502* (HITBC).

***Begonia handelii*** var. ***prostrata*** (Irmsch.) Tebbitt

**Laos**: Xiangkhoang: *A.F.G. Kerr 21772* (K).

***Begonia lancangensis*** S.H. Huang

**Laos**: Luang Namtha: *Tan et al. L0055* (HITBC).

***Begonia palmata*** D. Don

**Laos**: Houaphan: *E. Poilane 1938* (P), *E. Poilane 2002* (P); Khammouan: *M.F. Newman LAO1429* (E); Xiangkhoang: J. Delacour (P).

***Begonia quadripetiolata*** Aver. et H. Q. Nguyen

**Laos**: Vientiane: *L. Averyanov et al. LA-VN1510/1* (holotype LE01049395, isotype LE01049479).

***Begonia sizemoreae*** Kiew

**Laos**: Phongsaly: *Tan et al. L0559* (HITBC).

***Begonia siamensis*** Gagnep.

**Laos**: Attapu: *F.J. Harmand 1387* (P); Phongsaly: *Tan et al. L0476* (HITBC; HNL), *Tan et al. L0416* (HITBC).

***Begonia silletensis*** subsp. ***mengyangensis*** M.C. Tebbitt & K.Y. Guan

**Laos**: Luang Namtha: *Tan et al. L0095* (HITBC; HNL).

Begonia sect. Reichengeimia

***Begonia hymenophylla*** Gagnep.

**Laos**: Champasak: *C. Thorel 2358* (P), *C. Thorel* 2958 (P).

***Begonia hypoleuca*** Y.H. Tan & H.B. Ding

**Laos**: Luang Namtha: *Tan et al. L0792* (holotype HITBC!).

Begonis sect. Tetraphila

***Begonia afromigrata*** J.J. de Wilde

**Laos**: Muang Awn: *Kerr 20938* (BM, K, L); Vientiane: *Rodda*, *Simonsson MR106* (TO), *Rodda*, *Simonsson MR107* (FI).

## Supplementary Material

XML Treatment for Begonia laotica

XML Treatment for Begonia hypoleuca

XML Treatment for Begonia augustinei

XML Treatment for Begonia dryadis

XML Treatment for Begonia lancangensis

XML Treatment for Begonia sizemoreae

XML Treatment for Begonia silletensis subsp. mengyangensis
